# The Department of Modern Nursing

**Published:** 1915-01-16

**Authors:** 


### THE DEPARTMENT OF MODERN NURSING.*

### VII.—The Middlesex Hospital.

For some years past the Middlesex Hospital has been undergoing a wide reconstruction and development. The beds, which in 1901 numbered 308, now total 408, and the nursing staff has risen from 138 to 168. Within recent years the cancer wing has been completely rebuilt and organised as a separate department, with research laboratories attached; a new out-patients' department has been built; a new Nurses' Home, which the staff has already outgrown, has been added; a laundry has been built at Hendon, a convalescent home at Clacton; a new light department has been equipped, and some fine new wards secured. A new pathological department, the gift of Sir John Bland Sutton, is already rising in the background of the hospital. In every part of the institution growth has taken place and further developments are in prospect. The spirit of gentle humanity set stirring within the walls by former administrators is still one of its most conspicuous features, and those who are privileged to share in the work are still animated by the old loyalty to each other and to the best traditions of the hospital. To make part of the nursing staff in times of change, and when new currents of thought and new methods are on their trial, is to be preserved from any danger of stagnation and to be given uncommon opportunities for enlarging the experience and exhibiting the qualities. It is under such conditions that women of force and character come to the front.

### The Raw Probationer.

Among other innovations at the Middlesex the formation of a preliminary school is in contemplation. At present its inauguration is delayed by exigencies of space. But when the hospital obtains possession of the new plot of ground for which it has been long in treaty the preliminary school will come into being, together with a much-needed extension of the research department and other improvements. Probationers are received from the age of twenty-two, though no hard-and-fast rule debars the lady superintendent from admitting promising girls somewhat earlier if she thinks fit. The system of engagement is somewhat unusual. After a month's probation selected candidates serve for a probationary year, during which period they receive a salary of £12, but provide their own uniform. During this first year the probationers go through a course of lectures in general nursing, delivered by the home sister, and one in invalid cookery, delivered at the hospital by a County Council teacher. No examinations take place during this early period. At the end of the first year all subjects are tested by examination, and should the probationer fail in these tests "she will not be retained in the service of the hospital, except at the discretion of the nursing committee." This seems to us a very good working arrangement, entirely fair to the candidate. The examinations are not difficult, and should they reveal marked want of intelligence or care on the part of the examinee, the committee are enabled to dispense with the services of pupils who could never be trained into good nurses. On the other hand, should failure result from nervousness or from previous want of opportunities, rather than from want of general intelligence, the committee have powers to take into consideration the general level of the candidate's work, and give her a chance of improving later on. On entering the hospital an examination number is assigned to the candidate, which she retains till the termination of her engagement, and an admirable system is in force for ensuring that every probationer is thoroughly instructed in the practical part of her work. She receives at the beginning of her course a chart containing a list of fifty-five items, compiled with considerable skill, to enumerate all the practical processes in which the nurse ought to acquire facility.

### The Training Scheme.

Each sister as she teaches the nursing points mentioned in the left-hand column marks the same in the column descriptive of her ward—Surgical, Medical, etc. One stroke (/) signifies that the probationer has been shown, but is not perfected. A × means that the probationer has been taught and is proficient in that point. The sister placing a × inserts her initials in the right-hand column. The training scheme provides for each probationer to remain three months in a medical ward and three months in a surgical ward alternately. At the conclusion of her work in each ward the paper recording the nurse's progress, which is in her own keeping, is deposited for inspection in the matron's office. By this method an irrefragable proof is afforded of the nurse's proficiency in every practical nursing detail, and any omission in the training, however slight, at once springs into notice and can be repaired before the final examination. Monthly reports on each probationer are furnished by the sister of the ward, and these are filed away together, and collectively present a complete record of the nurse's progress through the hospital. The same conditions apply to paying probationers, who are charged 30 guineas for the first year's course.

### The Nurse's Course.

The probationer who passes creditably through her first year is recommended by the lady superintendent to the medical committee, and after "satisfying the resident medical officer that she is in sound health," commences her next course of training as "nurse." Second-year nurses normally have lectures on anatomy and physiology from Mr. Gordon Taylor and Dr. Voelcker. Third-year nurses attend four courses of lectures, one from Mr. Murray on surgical nursing, one from Dr. Wethered on medical nursing, one from Mr. Comyns Berkeley

* Previous articles have appeared in The Hospital of May 16 and 30, June 13, July 11 and 25, August 8.

on gynæcological nursing, and one from the lady superintendent on general nursing. The nurses have a commodious class and lecture room. Each course of lectures is followed by an examination, and, as before said, the services of nurses who fail to obtain 40 per cent. of marks in either anatomy or physiology may be dispensed with without further notice "at the discretion of the nursing committee." Nurses who pass are graded in three classes. There are three Fardon Memorial medals, gold (value £5), silver, and bronze, awarded each year to third-year nurses for proficiency in general training and good conduct. There is thus plenty of stimulus to mental exertion throughout the whole course of training, and in view of the extent to which heavy manual labours tend to divert the mind from intelligent appreciation of the medical and surgical work carried on in the wards, this is a precaution by no means to be despised.

During the second year senior probationers serve in the proportion of six months on day duty to three months on night duty. In the third year they advance to the status of staff nurses. The same arrangement of time holds good, usually three months on night and day duty alternately in the same ward. Thus at the conclusion of her third year a nurse would have had long experience of the work in at least eight wards, four during her first year and two during each subsequent year. The entire time of nurses during the first three years is spent among the patients, either in the wards or in the out-patient departments. None are employed on the domestic side or in the office. Nurses receive £18 in their second year, £20 in their third year, and £22 in their fourth year.

### Theatre and Special Work.

Two sisters and seven probationers in their second and third years are continuously employed in the theatre, the latter serving for a term of three months. Thus, twenty-eight nurses a year can be afforded thorough theatre training. In addition to this every nurse gets opportunities of accompanying her cases to the theatre and being present during the operation. Facilities are afforded the nurses for watching operations from above, and thus becoming familiar with the procedure. In the final year further opportunities are afforded the nurses for perfecting themselves in this branch of work.

The Middlesex is specially rich in the variety of work it embraces. The cancer wards are unique, not merely in the nursing of a special disease, but as an object lesson in dealing tenderly and scientifically with the dying. While the patients afford material for one of the most absorbing investigations of the day, their claims as individuals are generously recognised, and the atmosphere of these wards, where recovery is rare, is strangely cheerful and tranquil. The newly-equipped light department affords good training, and a special course of massage is available for a certain number of nurses "specially chosen by the lady superintendent," in their fourth year, supplementary lectures on anatomy being given to these pupils by the resident medical officer, and lessons in massage by the resident masseuse. It is also to be noted that any nurse who has completed her course of lectures and examinations can, on the recommendation of the lady superintendent, enter for a course of training in the maternity wards, and qualify for the Central Midwives Board examination. Lectures are given by Mr. Comyns Berkeley, by the house physician, and by Sister Annie Zunz. The fee for nurses of the hospital for sixteen weeks' training is £18.

### Nurses' Quarters and Recreation.

Every nurse after her first year has a good separate bedroom, and the community rooms are all well planned and prettily furnished. The patients' kitchen at the top of the building is a model of what such offices should be. Owing to considerations of space this kitchen is at present utilised to supply meals for others besides the nursing staff. Although the premises are fully adequate for the work carried on in them we cannot forbear regret that the exact statistics formerly available of the cost of the nurses' food can no longer be provided.

Nurses have two hours daily off duty, with three hours on Sunday and half a day every alternate week; they have three weeks' holiday in a year. Sisters have in addition three days or a week-end monthly, and twenty-eight days' holiday. Members of the nursing staff are nursed during illness, and receive their salary at the discretion of the weekly board. We learn with regret that the work of the private nursing staff has of late years dwindled to small proportions. Want of space is the main obstacle to maintaining a flourishing private staff, and the requirements of the hospital, which now claims an increased number of nurses, must necessarily come first. All the profits from the private nursing staff are carried to a separate pension fund account, from which retiring allowances are paid in proportion to length of service. Owing to this pension scheme the amount paid in pensions by the Middlesex Hospital exceeds that of any other London hospital except St. Bartholomew's, and amounted in 1911 to £763. This system will be considerably modified in the reorganisation of the private staff, which it is hoped will shortly take place, in order to meet the requirements of nurses who hesitate to engage themselves for an extended period of service.

### The Matron's Register.

The matron keeps a complete record of every nurse who receives training in the hospital. This record covers all particulars of her received before entering the hospital and enumerates how every day of her training was spent. It gives the result of all examinations, a list of all special subjects taken up, and a record of reports received from those under whom she worked. A space is left at the bottom for the entry of any items in her subsequent career with which the matron may become acquainted. This is in some important respects the best register of nurses with which we are familiar.

